# A semi-mechanism approach based on MRI and proteomics for prediction of conversion from mild cognitive impairment to Alzheimer’s disease

**DOI:** 10.1038/srep26712

**Published:** 2016-06-07

**Authors:** Haochen Liu, Xiaoting Zhou, Hao Jiang, Hua He, Xiaoquan Liu, Alzheimer’s Disease Neuroimaging Initiative, Michael W. Weiner, Paul Aisen, Ronald Petersen, Clifford R. Jack, William Jagust, John Q. Trojanowki, Arthur W. Toga, Laurel Beckett, Robert C. Green, Andrew J. Saykin, John Morris, Leslie M. Shaw, Zaven Khachaturian, Greg Sorensen, Maria Carrillo, Lew Kuller, Marc Raichle, Steven Paul, Peter Davies, Howard Fillit, Franz Hefti, Davie Holtzman, M. Marcel Mesulam, William Potter, Peter Snyder, Tom Montine, Ronald G. Thomas, Michael Donohue, Sarah Walter, Tamie Sather, Gus Jiminez, Archana B. Balasubramanian, Jennifer Mason, Iris Sim, Danielle Harvey, Matthew Bernstein, Nick Fox, Paul Thompson, Norbert Schuff, Charles DeCArli, Bret Borowski, Jeff Gunter, Matt Senjem, Prashanthi Vemuri, David Jones, Kejal Kantarci, Chad Ward, Robert A. Koeppe, Norm Foster, Eric M. Reiman, Kewei Chen, Chet Mathis, Susan Landau, Nigel J. Cairns, Erin Householder, Lisa Taylor-Reinwald, Virginia Lee, Magdalena Korecka, Michal Figurski, Karen Crawford, Scott Neu, Tatiana M. Foroud, Steven Potkin, Li Shen, Kelley Faber, Sungeun Kim, Kwangsik Nho, Lean Thal, Richard Frank, John Hsiao, Jeffrey Kaye, Joseph Quinn, Lisa Silbert, Betty Lind, Raina Carter, Sara Dolen, Beau Ances, Maria Carroll, Mary L. Creech, Erin Franklin, Mark A. Mintun, Stacy Schneider, Angela Oliver, Lon S. Schneider, Sonia Pawluczyk, Mauricio Beccera, Liberty Teodoro, Bryan M. Spann, James Brewer, Helen Vanderswag, Adam Fleisher, Daniel Marson, Randall Griffith, David Clark, David Geldmacher, John Brockington, Erik Roberson, Marissa Natelson Love, Judith L. Heidebrink, Joanne L. Lord, Sara S. Mason, Colleen S. Albers, David Knopman, Kris Johnson, Hillel Grossman, Effie Mitsis, Raj C. Shah, Leyla deToledo-Morrell, Rachelle S. Doody, Javier Villanueva-Meyer, Munir Chowdhury, Susan Rountree, Mimi Dang, Ranjan Duara, Daniel Varon, Maria T. Greig, Peggy Roberts, Yaakov Stern, Lawrence S. Honig, Karen L. Bell, Marilyn Albert, Chiadi Onyike, Daniel D’Agostino II, Stephanie Kielb, James E. Galvin, Brittany Cerbone, Christina A. Michel, Dana M. Pogorelec, Henry Rusinek, Mony J. de Leon, Lidia Glodzik, Susan De Santi, Kyle Womack, Dana Mathews, Mary Quiceno, P. Murali Doraiswamy, Jeffrey R. Petrella, Salvador Borges-Neto, Terence Z. Wong, Edward Coleman, Allan I. Levey, James J. Lah, Janet S. Cella, Jeffrey M. Burns, Russell H. Swerdlow, William M. Brooks, Steven E. Arnold, Jason H. Karlawish, David Wolk, Christopher M. Clark, Liana Apostolova, Kathleen Tingus, Ellen Woo, Daniel H.S. Silverman, Po H. Lu, George Bartzokis, Charles D. Smith, Greg Jicha, Peter Hardy, Partha Sinha, Elizabeth Oates, Gary Conrad, Neill R Graff-Radford, Francine Parfitt, Tracy Kendall, Heather Johnson, Oscar L. Lopez, MaryAnn Oakley, Donna M. Simpson, Martin R. Farlow, Ann Marie Hake, Brandy R. Matthews, Jared R. Brosch, Scott Herring, Cynthia Hunt, Anton P. Porsteinsson, Bonnie S. Goldstein, Kim Martin, Kelly M. Makino, M. Saleem Ismail, Connie Brand, Ruth A. Mulnard, Gaby Thai, Catherine Mc-Adams-Ortiz, Christopher H. van Dyck, Richard E. Carson, Martha G. MacAvoy, Pradeep Varma, Howard Chertkow, Howard Bergman, Chris Hosein, Sandra Black, Bojana Stefanovic, Curtis Caldwell, Ging-Yuek Robin Hsiung, Howard Feldman, Benita Mudge, Michele Assaly, Elizabeth Finger, Stephen Pasternack, Irina Rachisky, Dick Trost, Andrew Kertesz, Charles Bernick, Donna Munic, Kristine Lipowski, MASandra Weintraub, Borna Bonakdarpour, Diana Kerwin, Chuang-Kuo Wu, Nancy Johnson, Carl Sadowsky, Teresa Villena, Raymond Scott Turner, Kathleen Johnson, Brigid Reynolds, Reisa A. Sperling, Keith A. Johnson, Gad Marshall, Jerome Yesavage, Joy L. Taylor, Barton Lane, Allyson Rosen, Jared Tinklenberg, Marwan N. Sabbagh, Christine M. Belden, Sandra A. Jacobson, Sherye A. Sirrel, Neil Kowall, Ronald Killiany, Andrew E. Budson, Alexander Norbash, Patricia Lynn Johnson, Thomas O. Obisesan, Saba Wolday, Joanne Allard, Alan Lerner, Paula Ogrocki, Curtis Tatsuoka, Parianne Fatica, Evan Fletcher, Pauline Maillard, John Olichney, Owen Carmichael, Smita Kittur, Michael Borrie, T-Y Lee, Rob Bartha, Sterling Johnson, Sanjay Asthana, Cynthia M. Carlsson, Adrian Preda, Dana Nguyen, Pierre Tariot, Anna Burke, Nadira Trncic, Adam Fleisher, Stephanie Reeder, Vernice Bates, Horacio Capote, Michelle Rainka, Douglas W. Scharre, Maria Kataki, Anahita Adeli, Earl A. Zimmerman, Dzintra Celmins, Alice D. Brown, Godfrey D. Pearlson, Karen Blank, Karen Anderson, Laura A. Flashman, Marc Seltzer, Mary L. Hynes, Robert B. Santulli, Kaycee M. Sink, Leslie Gordineer, Jeff D. Williamson, Pradeep Garg, Franklin Watkins, Brian R. Ott, Henry Querfurth, Geoffrey Tremont, Stephen Salloway, Paul Malloy, Stephen Correia, Howard J. Rosen, Bruce L. Miller, David Perry, Jacobo Mintzer, Kenneth Spicer, David Bachman, Elizabether Finger, Stephen Pasternak, Irina Rachinsky, John Rogers, Dick Drost, Nunzio Pomara, Raymundo Hernando, Antero Sarrael, Susan K. Schultz, Laura L. Boles Ponto, Hyungsub Shim, Karen Ekstam Smith, Norman Relkin, Gloria Chaing, Michael Lin, Lisa Ravdin, Amanda Smith, Balebail Ashok Raj, Kristin Fargher

**Affiliations:** 1 Center of Drug Metabolism and Pharmacokinetics, China Pharmaceutical University, Nanjing, 210009, China; 2 UC San Francisco, San Francisco, CA 94107, USA.; 3 UC San Diego, La Jolla, CA 92093, USA.; 4 Mayo Clinic, Rochester, MN USA.; 5 UC Berkeley, Berkeley, San Francisco, USA.; 6 University of Pennsylvania, Philadelphia, PA 19104, USA.; 7 USC, Los Angeles, CA 90032, USA.; 8 UC Davis, Sacramento, CA, USA.; 9 Brigham and Women’s Hospital/Harvard Medical School, Boston, MA 02215 USA.; 10 Indiana University, Bloomington, IN 47405 USA.; 11 Washington University St. Louis, MO 63110 USA.; 12 Prevent Alzheimer’s Disease 2020, Rockville, MD 20850 USA.; 13 Siemens, Erlangen, Germany.; 14 Alzheimer’s Association, Chicago, IL 60631 USA.; 15 University of Pittsburg, Pittsburgh, PA 15213 USA.; 16 Cornell University, Ithaca, NY 14853 USA.; 17 Albert Einstein College of Medicine of Yeshiva University, Bronx, NY 10461 USA.; 18 AD Drug Discovery Foundation, New York, NY 10019 USA.; 19 Acumen Pharmaceuticals, Livermore, CA 94551, USA.; 20 Northwestern University, Chicago, IL 60611 USA.; 21 National Institute of Mental Health, Bethesda, MD 20892 USA.; 22 Brown University, Providence, RI 02912 USA.; 23 University of Washington, Seattle, WA 98195, USA.; 24 University of London, London, UK.; 25 UCLA, Torrance, CA 90509, USA.; 26 University of Michigan, Ann Arbor, MI, 48109-2800, USA.; 27 University of Utah, Salt Lake City, UT, 84112, USA.; 28 Banner Alzheimer’s Institute, Phoenix, AZ 85006, USA.; 29 UUC Irvine, Orange, CA 92868, USA.; 30 Johns Hopkins University, Baltimore, MD 21205 USA.; 31 Richard Frank Consulting, USA.; 32 National Institute on Aging, Baltimore, Maryland, USA.; 33 Oregon Health and Science University, Portland, OR 97239 USA.; 34 University of Alabama, Birmingham, AL USA.; 35 Mount Sinai School of Medicine, New York, NY USA.; 36 Rush University Medical Center, Chicago, IL 60612, USA.; 37 Baylor College of Medicine, Houston, TX, USA.; 38 Wien Center, Miami Beach, FL 33140 USA.; 39 Columbia University Medical Center, New York, NY USA.; 40 New York University, New York, NY USA.; 41 University of Texas Southwestern Medical School, Galveston, TX 77555 USA.; 42 Duke University Medical Center, Durham, NC USA.; 43 Emory University, Atlanta, GA, 30307, USA.; 44 University of Kansas Medical Center, Kansas City, Kansas, USA.; 45 University of Kentucky, Lexington, KY, USA.; 46 Mayo Clinic, Jacksonville, Florida, USA.; 47 University of Rochester Medical Center, Rochester, NY 14642, USA.; 48 Yale University School of Medicine, New Haven, CT, USA.; 49 McGill Univ. Montreal-Jewish General Hospital, Montreal, PQ H3A 2A7, Canada.; 50 Sunnybrook Health Sciences, Toronto, ON, Canada.; 51 U.B.C. Clinic for AD & Related Disorders, Vancouver, BC Canada.; 52 Cognitive Neurology - St. Joseph’s, London, ON, Canada.; 53 Cleveland Clinic Lou Ruvo Center for Brain Health, Las Vegas, NV 89106 USA.; 54 Premiere Research Inst (Palm Beach Neurology), W Palm Beach, FL USA.; 55 Georgetown University Medical Center, Washington, DC 20007 USA.; 56 Stanford University, Stanford, CA 94305, USA.; 57 Boston University, Boston, Massachusetts USA.; 58 Howard University, Washington, DC 20059 USA.; 59 Case Western Reserve University, Cleveland, OH 44106 USA.; 60 Neurological Care of CNY, Liverpool, NY 13088 USA.; 61 St. Joseph’s Health Care, London, ON N6A 4H1, Canada.; 62 Dent Neurologic Institute, Amherst, NY 14226 USA.; 63 Ohio State University, Columbus, OH 43210 USA.; 64 Albany Medical College, Albany, NY 12208 USA.; 65 Hartford Hospital Olin Neuropsychiatry Research Center, Hartford, CT 06114 USA.; 66 Dartmouth-Hitchcock Medical Center, Lebanon, NH, USA.; 67 Wake Forest University Health Sciences, Winston-Salem, NC, USA.; 68 Medical University South Carolina, Charleston, SC 29425 USA.; 69 Nathan Kline Institute, Orangeburg, NY USA.; 70 University of Iowa College of Medicine, Iowa City, IA 52242 USA.; 71 University of South Florida: USF Health Byrd Alzheimer’s Institute, Tampa, FL 33613 USA.

## Abstract

Mild cognitive impairment (MCI) is a precursor phase of Alzheimer’s disease (AD). As current treatments may be effective only at the early stages of AD, it is important to track MCI patients who will convert to AD. The aim of this study is to develop a high performance semi-mechanism based approach to predict the conversion from MCI to AD and improve our understanding of MCI-to-AD conversion mechanism. First, analysis of variance (ANOVA) test and lasso regression are employed to identify the markers related to the conversion. Then the Bayesian network based on selected markers is established to predict MCI-to-AD conversion. The structure of Bayesian network suggests that the conversion may start with fibrin clot formation, verbal memory impairment, eating pattern changing and hyperinsulinemia. The Bayesian network achieves a high 10-fold cross-validated prediction performance with 96% accuracy, 95% sensitivity, 65% specificity, area under the receiver operating characteristic curve of 0.82 on data from the Alzheimer’s Disease Neuroimaging Initiative (ADNI) database. The semi-mechanism based approach provides not only high prediction performance but also clues of mechanism for MCI-to-AD conversion.

Alzheimer’s disease (AD), the most common form of dementia, is characterized by progressive neurodegenerative disorder^1^. 36 million people worldwide are affected by AD and the number is expected to almost triple by 2050^2^. Many evidences indicate that AD has a years to decade preclinical period followed by a precursor phase termed as mild cognitive impairment (MCI)^3^. As new treatments are likely to be most effective at the early stages of AD, it is greatly urgent to track patients with MCI who will develop AD^4^,^5^.

Several sensitive imaging modalities such as structural magnetic resonance imaging (MRI) and positron emission tomography (PET) have been developed^5^. A number of previous researches have reported that MRI biomarkers can be used to predict the probability of conversion^6^,^7^,^8^. However, because some of structural changes may not be detected at visual inspection until MCI patients have converted to AD, predictions using MRI biomarkers only may not be accurate enough for application in the routine clinical setting or clinical drug trials^3^,^5^. Previous researches show that combined markers such as MRI and cerebrospinal fluid (CSF) biomarkers can improve the prediction accuracy^5^,^9^. But CSF sample collection requires lumbar puncture which is too invasive to be used as a routine clinical examination. As damage to the blood-brain barrier may occur in AD, this may increase movement of proteins between the brain and the blood^10^. It is therefore possible that AD and its precursor, MCI, may be associated with the variation of biomarkers detectable in plasma^11^. Recent work has demonstrated the possibility of predicting MCI-to-AD conversion based on plasma markers^12^. In addition, blood sample is more accessible and suitable for repeated collecting. These make plasma-based biomarkers promising for prediction of conversion from MCI to AD.

While the highly sensitive markers are beneficial on the conversion prediction, advanced machine learning methods can further improve the reliability of approaches. Machine learning is the study of algorithms and computational techniques that use previous examples in the form of multivariate datasets to help make future predictions^13^. A number of machine learning methods such as support vector machines (SVM) and logistic regression (LR) have been used to predict the conversion from MCI to AD^5^,^8^. Compared with the traditional data-driven machine learning methods, Bayesian network has unique advantages that it can quantify the causal relationships between the markers, visualize these relationships by the structure of network, and conduct the prediction task based on the causal relationships^14^. These attractive characteristics make Bayesian network a semi-mechanism method. On one hand, the semi-mechanism nature of Bayesian network can improve our understanding of conversion mechanism. On the other hand, because of the complex etiology and multiple pathogenesis of AD, the conversion from MCI to AD is affected by many uncertain factors which makes its prediction a complicated issue^15^. Bayesian network is especially well-suited to handle the intricacies of the prediction because it is designed for representing stochastic events and conducting prediction tasks under uncertainty^16^,^17^.

Lots of lectures based on data-driven methods, such as neural network with self-organizing maps (SOM), are focused on improving the classification performance and they have showed good performance in the diagnosis task. However, the contribution of these methods on improving our understanding of MCI-to-AD conversion mechanism is limited. As the semi-mechanism nature of Bayesian network can provide causal relationships of markers, this paper proposes a semi-mechanism method based on the combination of Bayesian network and lasso regression for not only the high performance of MCI-to-AD conversion prediction but also improving our understanding the mechanism of the conversion. The data from Alzheimer’s Disease Neuroimaging Initiative (ADNI) is used to develop the model. However, ADNI contains more than 500 biomarkers (including MRI markers and plasma markers), many of which may not relate to MCI-to-AD conversion. Irrelevant biomarkers may interfere the causal relationships identification and reduce the performance of prediction method. Therefore, biomarkers selection should be performed before the conversion prediction. In this study, lasso regression is proposed to conduct the markers selection, which combines variable selection with an efficient computational procedure^18^. Previous works have shown that lasso regression can enhance the prediction performance of models based on high dimension data sets^19^,^20^,^21^. As such, the combination of Bayesian network and lasso regression is proposed not only to conduct the prediction task but also to improve understanding of the AD-to-MCI conversion mechanism. Moreover, after the conversion probability is calculated, a subgroup analysis is performed for comparing the network disruption of high-risk patients and low-risk patients.

## Results

### Biomarkers selection

**Figure 1:**
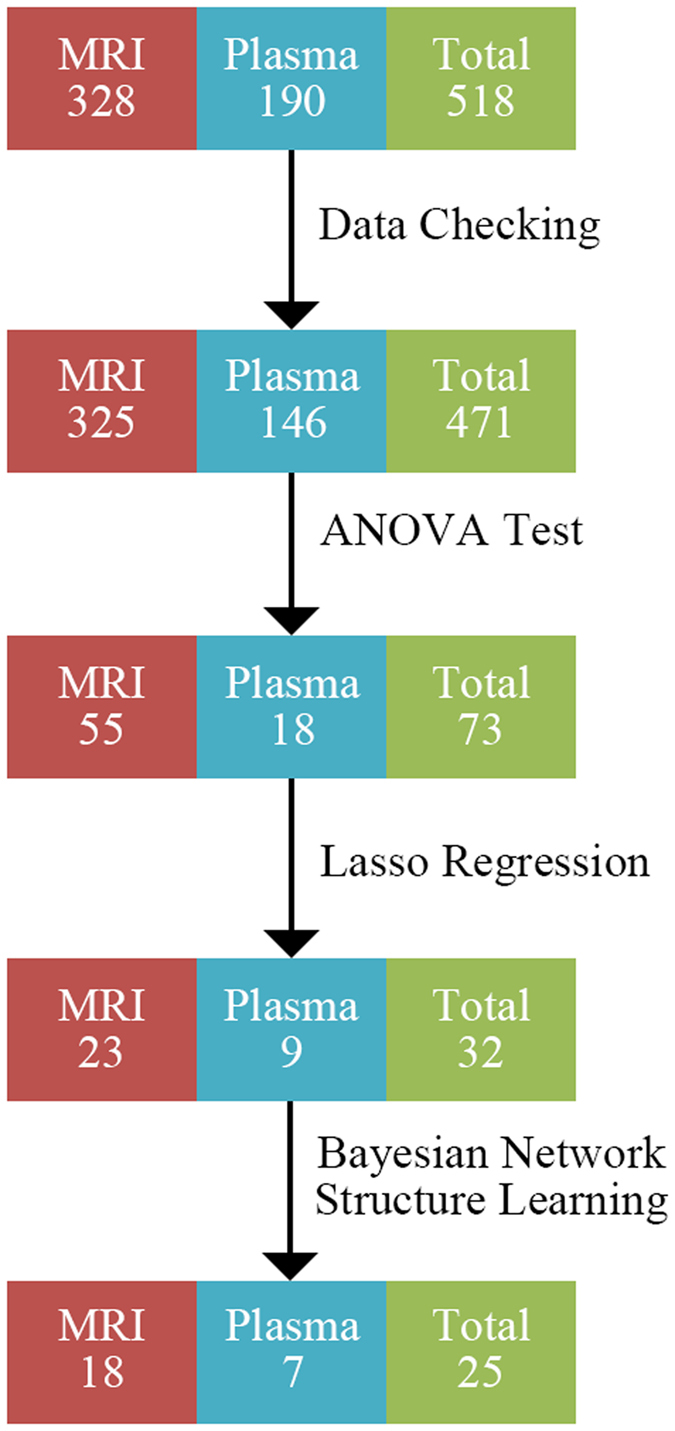
The process of markers selection.

In this section, the process of biomarkers selection is described. The dataset used in this study contains 518 biomarkers (328 MRI markers and 190 plasma markers). 45 biomarkers (1 MRI marker and 44 plasma markers) are deleted during data checking due to too many missing entries. 75 biomarkers (57 MRI markers and 18 plasma markers) with significant difference between converters and non-converters are identified by ANOVA test. 34 biomarkers (25 MRI markers and 9 plasma markers) related to Alzheimer’s disease assessment scale (ADAS-cog) are selected by lasso regression. 7 biomarkers (5 MRI markers and 2 plasma markers) are eliminated during Bayesian network structure learning because they fail to connect to the Bayesian network. In addition, as 2 MRI markers are labeled as “unknown”, they are also eliminated. Finally, 25 biomarkers (18 MRI markers and 7 plasma markers) are selected for conversion prediction. The process of biomarkers identification is summarized in Fig. 1. The list of selected biomarkers is shown in Table 1.

### Structure and performance of Bayesian network

**Figure 2:**
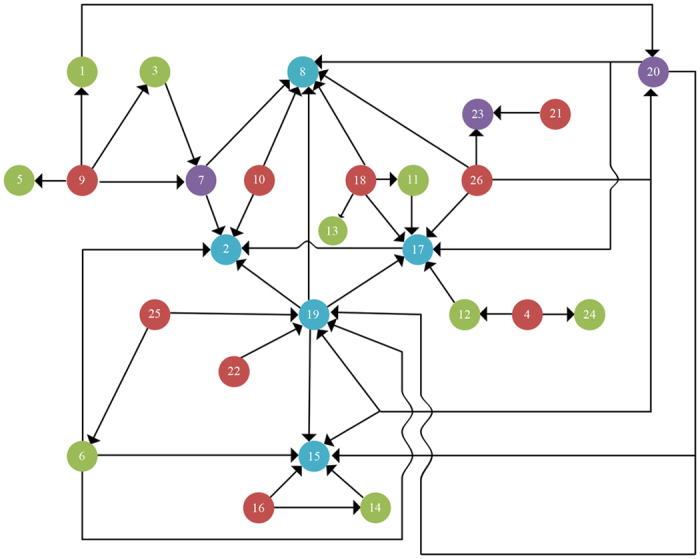
The structure of Bayesian network. It contains 26 nodes and 43 arcs. The nodes in order are: ST109TS, ST111CV, ST114TA, ST11SV, ST121TA, ST30SV, ST31TA, ST40CV, ST49TA, ST52CV, ST56CV, ST70SV, ST72CV, ST83CV, ST83TA, ST88SV, ST91CV, ST99CV, AGRP, C-peptide, CRP, FGF-4, Fibrinogen, Insulin, MMP-10, and “Whether patients converts to AD or not”.

In this section, we present the results of Bayesian structure learning and the performance of conversion prediction. The Bayesian network structure obtained by max-min hill-climbing (MMHC) is given in Fig. 2. It contains 26 nodes and 43 arcs.

**Figure 3:**
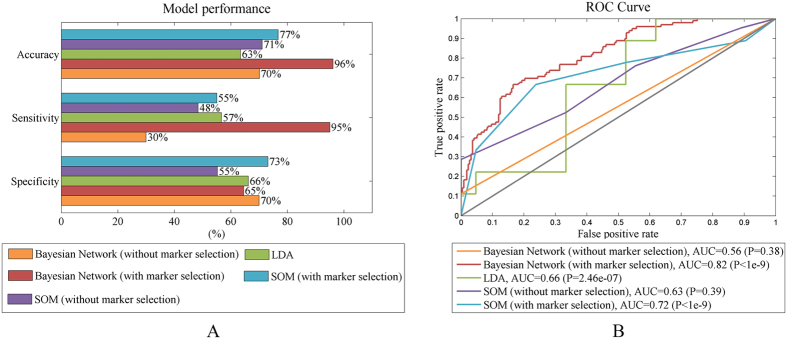
The performance of five different conversion prediction models. (**A**) The receiver operating characteristic (ROC) curve of Linear discriminant analysis (LDA), self-organizing map (SOM) (with or without markers selection) and Bayesian network (with or without markers selection). (**B**) The performance of LDA, SOM (with or without markers selection) and Bayesian network (with or without selection) measured by three parameters: accuracy, sensitivity, specificity. All these parameters are evaluated by 10-fold cross-validation.

In order to evaluate the performance of Bayesian network, a 10-fold cross-validation is performed to estimate its accuracy, sensitivity and specificity. Furthermore, the performance of Bayesian network is compared to the performances of linear discriminant analysis (LDA) and SOM. The performances of all these methods are evaluated by 10-fold cross-validation. The results are given in Fig. 3. The Fig. 3A shows that the accuracy and sensitivity of Bayesian network are higher than those of LDA and SOM with markers selection. In Fig. 3B, the area under receiver operating characteristic curve (AUC-ROC) of Bayesian network is much higher than that of LDA and SOM with marker selection. Moreover, to evaluate the performance of markers selection, we apply SOM and Bayesian network with or without markers selection and compare their performances. With markers selection, the classification performances of both SOM and Bayesian network are improved.

### Network disruption profile

**Figure 4:**
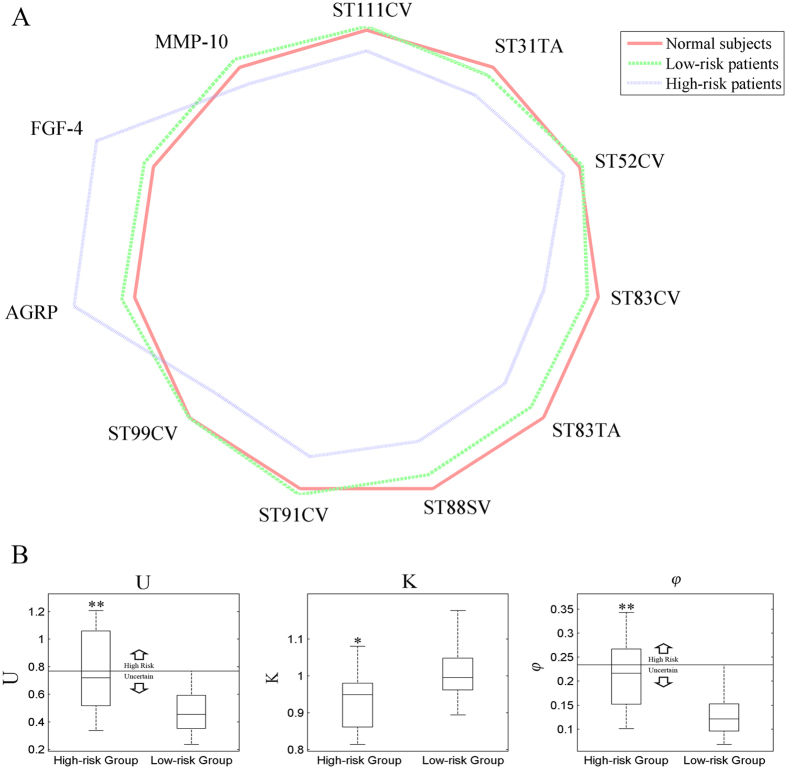
(**A**) Network disruption analysis of markers with significant difference between high-risk group and low-risk group. In normal state, the shape of radar graph is a regular polygon. With the shape deformation, the difference from normal state gets greater. (**B**) Box plot of parameters U, K, and 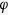. If the value of disruption parameters U and 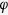 is beyond the horizontal lines in figures, the patient may have more conversion risk. *P < 0.05, **P < 0.01 vs low risk group.

According to the result of Bayesian network, a group of highest conversion probability patients (high-risk group, n = 11) and a group of lowest conversion probability patients (low-risk group, n = 48) are drawn from the dataset. 11 biomarkers have significant difference (P < 0.05, ANOVA test) between high-risk group and low risk group. The mini network balance map Fig. 4A) shows that the high-risk group may suffer from more severe network disruption than the low risk group. The network disruption parameters coincide with the mini network balance map. Parameters U and 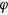 increase significantly in high risk group (P < 0.01, ANOVA test, shown in Fig. 4B) which may suggest that patients with greater U and 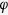 may have higher conversion risk.

## Discussion

In this study, we propose a semi-mechanism based Bayesian network to predict the conversion from MCI to AD. The proposed method has two contributions. Firstly, the proposed approach achieves relative high prediction performance. Secondly, as the Bayesian network can learn the causal relationships among biomarkers from the database, these causal relationships can provide some more insight into the mechanism of MCI-to-AD conversion.

The proposed model is compared to previous researches based on data-driven methods (Table 2). Comparing with LDA and SOM, Bayesian network has higher accuracy and sensitivity with markers selection. The high sensitivity of Bayesian network may lie in two points. On one hand, the semi-mechanism nature of Bayesian network may provide higher performance because it can learn causal relationships from data and combine these knowledge and data to conduct the prediction task^22^. On the other hand, plasma markers may be highly sensitive in conversion prediction^12^. Though the data-driven methods also achieved high performance, the Bayesian network still has its unique advantage. The structure of Bayesian network may contain the causal relationships of markers which makes it a semi-mechanism method and provide more information beyond the performance of classification.

In addition, with markers selection, the classification performance of Bayesian network is improved. It suggests that Bayesian network should work with an appropriate marker selection strategy. In another words, without markers selection, Bayesian network may produce false positive causal relationships which may not only decrease the performance but also mislead the MCI-to-AD conversion mechanism investigation. Therefore, combining Bayesian network and lasso marker selection strategy is very helpful in improving understanding the conversion mechanism and classification performance.

The semi-mechanism nature of Bayesian network is beneficial on investigating the mechanism of conversion. Structure of Bayesian network shows that 6 markers including volume of left middle temporal, cortical thickness average of right entorhinal, volume of right inferior temporal, AGRP, c-peptide, and fibrinogen may be related to the conversion directly. Our result, that destruction of entorhinal is associated with MCI-to-AD conversion, is consistent with previous research^23^. Previous researches had also reported the variations of temporal, c-peptide level, and fibrinogen level in AD patients^19^,^24^,^25^. However, our results suggest that these changes may have happened at MCI stage. It indicates that the conversion from MCI to AD may start with destruction of temporal, entorhnal, increased level of AGRP, c-peptide, and fibrin^25^.

Bayesian network identifies the variations of above six markers caused by MCI-to-AD conversion directly. But some of these changings may not be the key factors in the conversion. Therefore a reanalysis based on the results of Bayesian network is performed to identify the major factors. The subgroups network disruption profile suggests that the progress MCI patient may suffer from more severe network disruption than stable MCI patients. Network disruption may be related to the marker panel including 11 markers. The six markers identified by Bayesian network and marker panel related network disruption share three markers: Cortical Thickness of Entorhinal, Volume of Temporal and AGRP. These three markers may be the key factors in the conversion. In other word, they might be attributed to the warming signals of conversion. Previous researches showed that the destruction of entorhinal and temporal is associated with verbal memory impairment and verbal memory impairment might be the warming indicator of MCI-to-AD conversion^26^. In addition, clinical researches have reported that AD patients have greater preference for high-fat and sweet food than normal groups. However, our results suggested that such change in eating pattern may have happened at MCI stage^27^,^28^, as the elevated level of AGRP, an orexigenic peptide, in high-risk patients may increase the preference for a high fat diet^29^.

The crosstalk between cerebral destruction and plasma markers alteration revealed by Bayesian network can provide more clues for the mechanism of conversion. The crosstalk between C-peptide and cerebral destruction may play a vital role in the MCI-to-AD conversion. C-peptide is a measure of insulin secretion. Elevated C-peptide level represents high peripheral insulin secretion. It is reported that high peripheral insulin secretion can increase the risk of AD. Because high level peripheral insulin secretion impairs amyloid clearance by inhibiting brain insulin production which is a beneficial effect on amyloid clearance^30^. Bayesian network suggests that C-peptide may be related to the destruction of middle temporal, entorhinal, and inferior temporal. It suggests that amyloid may mainly aggregate in the above three regions at the MCI stage which may aggravate their damage. As all above three regions are involved in verbal memory, high level of C-peptide may impair to verbal memory which was confirmed by previous works^26^,^31^,^32^,^33^.

In summary, the analysis of Bayesian network shows that the conversion from MCI to AD may start with multiple pathological changes such as verbal memory impairment, vascular abnormalities, hyperinsulinemia and eating pattern change. In this study, a high performance semi-mechanism based approach is developed to predict the conversion from MCI to AD by combining MRI and plasma markers. The semi-mechanism based approach provides not only high performance prediction but also more insight into the mechanism of conversion from MCI to AD.

## Subject and Method

### Subject

#### Patients

In this study, the following criteria are used to select subjects for model developing:

- Patients with baseline MRI scan records
- Patients with baseline plasma-based biomarker data
- Patients with baseline ADAS-cog scores
- Patients with MCI due to Alzheimer’s disease
- Patients with diagnosis records which can be used to determine whether they convert from MCI to AD in 18 months

Finally, a data set with complete imaging, plasma-based biomarkers, ADAS data is drawn from ADNI including 316 MCI patients (99 converters and 217 non-converters). The demographic information of subjects is given in Table 3.

#### Imaging biomarkers

Imaging data in this study is obtained from dataset UCSF—Cross-Sectional FreeSurfer (FreeSurfer Version 4.3). The dataset is available at https://ida.loni.usc.edu/pages/access/studyData.jsp. In this dataset, all scans were acquired on 1.5 T MRI scanners. The imaging data were processed and analyzed with FreeSurfer 4.3 by the UCSF team. The dataset includes 328 MRI biomarkers which can be grouped into 5 categories: average cortical thickness, standard deviation in cortical thickness, the volumes of cortical parcellations (based on regions of interest automatically segmented in the cortex), the volumes of specific white matter parcellations, and the total surface area of the cortex. Details of the analysis procedure are available at http://adni.loni.ucla.edu/research/mripost-processing/.

#### Plasma-based biomarkers

The plasma-based biomarker data is obtained from dataset Biomarkers Consortium Plasma Proteomics Project RBM multiplex data. The data is available at https://ida.loni.usc.edu/pages/access/studyData.jsp. The data was acquired by analyzing a subset of plasma samples from the ADNI cohort in a 190 analyte multiplex immunoassay panel. The panel, referred to as the human discovery map, was developed on the Luminex xMAP platform by Rules-Based Medicine (RBM) to contain proteins previously reported in the literature to be altered as a result of cancer, cardiovascular disease, metabolic disorders and inflammation. Details of the assay technology and validation has been described elsewhere (http://adni.loni.ucla.edu/wp-content/uploads/2010/11/BC_Plasma_Proteomics_Data_ Primer.pdf).

### Method

**Figure 5:**
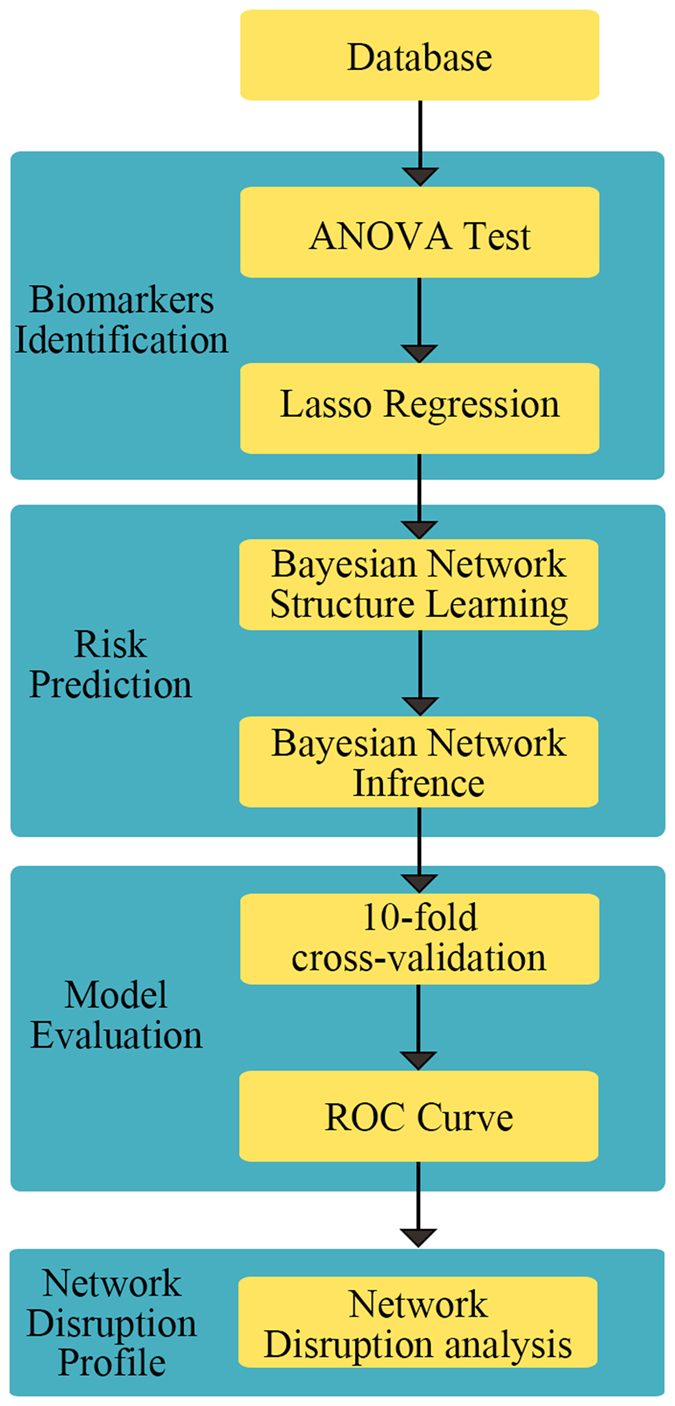
The machine learning framework.

Considering that ADNI contains more than 500 biomarkers, it is essential to select the more predictive biomarkers to obtain a parsimonious model and avoid the classifier suffering overfitting. Then the Bayesian network is established based on the causal relationships among selected markers for predicting the AD-to-MCI conversion. Finally, a reanalysis of Bayesian network results is performed to profile the network disruption of the patients with highest probability of converting to AD and those with lowest probability. The framework is summarized in Fig. 5.

#### Biomarkers selection

Biomarkers selection includes two stages. At the first stage, ANOVA test is employed to screen biomarkers with significant difference (P < 0.05) between converters and non-converters. At the second stage, lasso regression is used to filter biomarkers related to ADAS-cog from the selected biomarkers at the first stage.

Lasso regression is a popular technique for feature selection which can continuously shrinks coefficients^34^. It drops biomarkers by shrinking some of coefficients to zero. In this study, a Least Angle Regression (LARS) algorithm is used to solve lasso^35^.

#### Bayesian network

Considering that the causal relationships among the selected markers may remain unknown, a Bayesian network structure learning algorithm termed as the max-min hill-climbing (MMHC) is employed to learn the causal relationships among the selected markers. MMHC algorithm is a hybrid method, using concepts and techniques from both constraint-based approaches and score-based approaches, which can achieve high quality in structure learning^36^. After the Bayesian network is learned from data, the most popular Bayesian network inference algorithm named junction tree is employed to acquire the conversion prediction^37^.

#### Model evaluation

In this study, the receiver operating characteristic (ROC) curve is used to evaluate the performance of Bayesian network. The ROC, which has become established as an important tool for classifier evaluation, is a graph of true positive rate (TPR) against false positive rate (FPR) at various operating points as a decision threshold^38^. The area under the ROC curve (AUC) is a measure of predictive ability^39^. Moreover, three parameters termed as accuracy (number of correctly classified samples divided by the total number of samples), sensitivity (the number of correctly classified converters divided by the total number of converters) and specificity (the number of correctly classified non-converters divided by the total number of non-converters) are calculated and evaluated by 10-fold cross-validation for a further measurement for the model performance^40^.

#### Network disruption analysis

To get more insight into the mechanism of the conversion, a reanalysis of Bayesian network results is performed using a mathematic method for evaluating the disruption of biology network which was proposed in our previous research^41^. In this study, subjects are divided into two subgroups high risk group and low risk group according to the results of Bayesian network and a mini network balance model is developed to evaluate the network disruption for both high-risk group and low-risk group. The network disruption comparison between these two subgroups may provide more insight into the mechanism of AD-to-MCI conversion.

The mini network balance model contains three parameters U, K, and 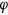. U is response to both consistency variation and inconsistency variation comprehensively. K responds to multi-marker consistency variation. 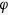 is response to the multi-marker inconsistency variation. These three parameters can be calculated as below:


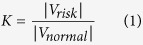



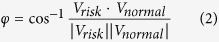



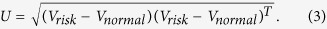


Let 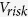 be the state vector of patients with conversion risk and 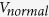 be the state vector of normal control group.

## Additional Information

**How to cite this article**: Liu, H. *et al.* A semi-mechanism approach based on MRI and proteomics for prediction of conversion from mild cognitive impairment to Alzheimer's disease. *Sci. Rep.* **6**, 26712; doi: 10.1038/srep26712 (2016).

## Figures and Tables

**Table 1:** List of selected markers.

Number	Abbreviation	Marker	Source
1	ST109TS	Cortical Thickness SD of Right Posterior Cingulate	MRI
2	ST111CV	Volume of Right Precuneus	MRI
3	ST114TA	Cortical Thickness Average of Right Rostral Middle Frontal	MRI
4	ST11SV	Volume (WM Parcellation) of Left Accumbens Area	MRI
5	ST121TA	Cortical Thickness Average of RightTransverseTemporal	MRI
6	ST30SV	Volume (WM Parcellation) of Left Inferior Lateral Ventricle	MRI
7	ST31TA	Cortical Thickness Average of Left Inferior Parietal	MRI
8	ST40CV	Volume (Cortical Parcellation) of Left Middle Temporal	MRI
9	ST49TA	Cortical Thickness Average of Left Postcentral	MRI
10	ST52CV	Volume (Cortical Parcellation) of Left Precuneus	MRI
11	ST56CV	Volume (Cortical Parcellation) of Left Superior Frontal	MRI
12	ST70SV	Volume (WM Parcellation) of Right Accumbens Area	MRI
13	ST72CV	Volume (Cortical Parcellation) of superior temporal sulcus	MRI
14	ST83CV	Volume (Cortical Parcellation) of Right Entorhinal	MRI
15	ST83TA	Cortical Thickness Average of Right Entorhinal	MRI
16	ST88SV	Volume (WM Parcellation) of Right Hippocampus	MRI
17	ST91CV	Volume (Cortical Parcellation) of Right Inferior Temporal	MRI
18	ST99CV	Volume (Cortical Parcellation) of Right Middle Temporal	MRI
19	AGRP	Agouti-Related Protein (AGRP)	Plasma
20	–	C-peptide	Plasma
21	CRP	C-Reactive Protein (CRP)	Plasma
22	FGF-4	Fibroblast Growth Factor 4 (FGF-4)	Plasma
23	–	Fibrinogen	Plasma
24	–	Insulin (uIU/mL)	Plasma
25	MMP-10	Matrix Metalloproteinase-10 (MMP-10)	Plasma
26	–	Whether patients converts to AD or not	–

**Table 2:** Comparisons to other methods.

Research	Included components	Sample size	Results
Bayesian network (with marker selection, this study)	MRI + plasma	365	Accuracy = 96%
			Sensitivity = 95%
			Specificity = 63%
			AUC = 0.82
Bayesian network (without marker selection, this study)	MRI + plasma	365	Accuracy = 70%
			Sensitivity = 30%
			Specificity = 70%
			AUC = 0.56
neural network with self-organizing maps (SOM) (with marker selection, this study)	MRI + plasma	365	Accuracy = 77%
			Sensitivity = 55%
			Specificity = 73%
			AUC = 0.72
SOM (without marker selection, this study)	MRI + plasma	365	Accuracy = 71%
			Sensitivity = 48%
			Specificity = 55%
			AUC = 0.63
Linear discriminant analysis (LDA) (with marker selection, this study)	MRI + plasma	365	Accuracy = 63%
			Sensitivity = 57%
			Specificity = 66%
			AUC = 0.66
Linear discriminant analysis (LDA)^8^	MRI	405	Accuracy = 68%
			Sensitivity = 67%
			Specificity = 69%
Gularized logistic regression (RLR)^42^	CSF	335	Accuracy = 53%
			Sensitivity = 31%
			Specificity = 73%
Domain transfer learning^43^	PET	99	Accuracy = 71%
			Sensitivity = 76%
			Specificity = 67%
			AUC = 0.74
Multi-task Linear Programming Discriminant (MLPD)^44^	MRI + PET	202	Accuracy = 67%
			Sensitivity = 68%
			Specificity = 67%
Logistic regression models^5^	MRI + PET + CSF	97	Accuracy = 72%
low density separation (LDS)^40^	MRI + age + cognitive score	394	Accuracy = 82%
			Sensitivity = 87%
			Specificity = 74%
			AUC = 0.9

**Table 3:** Subjects demographic information.

	MCI	Converters	Non-Converters
Number	316	99	217
Age	74.68 ± 7.23	74.72 ± 7.25	74.67 ± 7.25
Gender (male/female)	206/110	58/41	148/69
ADAS-cog (85 points total)	18.63 ± 6.36	22.36 ± 4.56	16.94 ± 4.84
